# Communicating the promise, risks, and ethics of large-scale, open space microbiome and metagenome research

**DOI:** 10.1186/s40168-017-0349-4

**Published:** 2017-10-04

**Authors:** Daria Shamarina, Iana Stoyantcheva, Christopher E. Mason, Kyle Bibby, Eran Elhaik

**Affiliations:** 1 Department of Molecular Biology and Biotechnology, University of Sheffield, Sheffield, S10 2TN UK; 2 Department of Physiology and Biophysics, Weill Cornell Medicine, New York, NY 10021 USA; 3 The HRH Prince Alwaleed Bin Talal Bin Abdulaziz Alsaud Institute for Computational Biomedicine, New York, NY 10021 USA; 4 The Feil Family Brain and Mind Research Institute, Weill Cornell Medicine, New York, NY 10021 USA; 5 University of Notre Dame Department of Civil and Environmental Engineering and Earth Sciences, University of Notre Dame, Notre Dameᅟ, IN 46556 USA; 6 Department of Animal and Plant Sciences, University of Sheffield, Sheffield, S10 2TN UK

**Keywords:** Microbiome, Metagenome, Built environment, Public, MetaSUB, Concerns, Ethics

## Abstract

The public commonly associates microorganisms with pathogens. This suspicion of microorganisms is understandable, as historically microorganisms have killed more humans than any other agent while remaining largely unknown until the late seventeenth century with the works of van Leeuwenhoek and Kircher. Despite our improved understanding regarding microorganisms, the general public are apt to think of diseases rather than of the majority of harmless or beneficial species that inhabit our bodies and the built and natural environment. As long as microbiome research was confined to labs, the public’s exposure to microbiology was limited. The recent launch of global microbiome surveys, such as the Earth Microbiome Project and MetaSUB (Metagenomics and Metadesign of Subways and Urban Biomes) project, has raised ethical, financial, feasibility, and sustainability concerns as to the public’s level of understanding and potential reaction to the findings, which, done improperly, risk negative implications for ongoing and future investigations, but done correctly, can facilitate a new vision of “smart cities.” To facilitate improved future research, we describe here the major concerns that our discussions with ethics committees, community leaders, and government officials have raised, and we expound on how to address them. We further discuss ethical considerations of microbiome surveys and provide practical recommendations for public engagement.

## Background

Until recently, microbial communities have typically been studied in research facilities, largely out of the public eye. The rapid advent of high-throughput molecular techniques prompted a dramatic increase in the ability to study these communities in the human body [1] and over a wider range of habitats including urban [2, 3] and indoor [4–7] environments. These studies have provided valuable insight about the amount and type of bacteria in our environment and their ecology, source [8], and effects on our health [9] and behavior [10].

The vital contribution of microorganisms to our environment and health calls for large-scale efforts to chart our indoor and immediate outdoor microbiome environments. Consequently, over the past decade [11] several mapping efforts charting public restrooms [9], apartments [10], university classrooms and office buildings [11], hospitals [12], museum artifacts [13], dust [14], metropolitan subways [2, 15–17] were launched. Reviews regarding the scientific findings of these studies investigating the “built environment” have recently been published [18]. One notable initiative, the MetaSUB project, originated to capture a city-scale molecular profile of DNA collected in New York [2]. The project has now grown into a consortium encompassing 72 major worldwide cities. Such studies challenge our perception of public health, safety, and privacy in urban environments, and seek to advance new strategies to protect our growing society, such as the design of “smart cities” that convey early warnings for potential epidemics and genetically protected infrastructure. While some studies aim to involve public members and educate them about their internal and external microbial environments, the growing number of community-level microbiome studies could inadvertently bring a negative image of microorganisms that would foster public fear [19] of such research and impede future microbiome investigations.

Building on our years of experience in the International MetaSUB Consortium [20–23], we compiled a list of concerns—all raised during our discussions with ethics committees, community leaders, and government officials—and address them. We also discuss how technological advances may change these assessments and provide recommendations for public engagement in future studies. The concerns identified and explained here, and the recommendations for public engagement, can be used as guidelines and benefit microbiome and metagenome research.

## The most common concerns associated with the public impact of microbiome research

### Ostracizing sensitive populations

Since the earliest human microbiome project in 1683 when Antonie van Leeuwenhoek scraped “gritty matter” from between his teeth and visualized bacteria, research on human-associated microorganisms has focused on pathogens and the environments that allowed them to flourish [24], linking the presence of microorganisms and human contact with pathogenicity [25].

Microbiome studies are typically targeted, at least in part, at quantifying microbial biodiversity. Biodiversity of bacteria is measured in terms of taxonomy, diversity, evolutionary distance, their amount or frequency, and dynamics over time. Pathogenicity is related to, but not necessarily linked with biodiversity. Nonetheless, the presence of microorganisms, cleanliness, sanitation, and health quickly became embedded cross-culturally. These terms and themes have been used as a pejorative that stigmatized individuals, people, cultures, places, and cities [26–28]. “Dirt,” for instance, is a common pejorative in hygienic racism that has been applied to minorities, disadvantaged communities, and indigenous people who were then subjected to discrimination based on their hygiene and health [29]. For instance, due to poor housing conditions, Australian children living in remote indigenous communities experience high rates of increased transmission of infection compared to other Australian children. Racism and housing conditions are both associated with child health and with adult physical and mental health [29, 30]. That poverty, sanitation, and infection go hand in hand have thereby contributed to the stigmatization of sensitive populations. It follows that there is a well-placed concern that investigating the microbiome of a location may lead to its association with disease, to the detriment of its residents.

Recent studies, however, do not support the presumed association between biodiversity and pathogenicity. Microbiome studies can distinguish between ecologically varied diverse regions, which can be influenced by human biodiversity, and even reflected in the DNA left on ATM keypads [31], showing that the bacterial diversity is not generally related with pathogenicity or poverty. For example, in New York City, the Bronx was the most diverse borough both in terms of human and bacterial diversity [2]. Poverty rates in Manhattan are three quarters those of Brooklyn [32], but they have similar levels of bacterial diversity [2]. Governments are largely responsible for sanitation systems, particularly in large cities, and it is acknowledged that sanitary neglect is a population-wide hazard. Moreover, microorganisms are also produced by animals inhabiting the surrounding environment, like rats, dogs, and pigeons [2, 31]—and are thought to be among the major sources of zoonotic infections, such as *T. gondii*, in big cities [33]. The scientific community should thereby be cognizant of the language and its perception by the media and the general public. Interestingly, advances in epidemiology over the past two decades highlighted that “over-clean” environment pose a risk to human health, whereas exposure to little dirt (and most importantly, microorganisms) can be beneficial and even ward off disease [34, 35].

### Drawing the public away from transit systems

Due to high levels of human traffic, handles, seats, and even the air transport systems are often perceived to be biologically contaminated [36–38]. Evidence suggests that during an epidemic outbreak, individuals may alter their behavior to reduce their risk of infection. For example, during the 2009 H1N1 influenza, 16–25% of Americans avoided places where many people gathered, including public transportation [39]. Another example emerged during the 2014 Ebola outbreak [40] when the Centers for Disease Control and Prevention and World Health Organization recommended screening airport passengers arriving from countries with Ebola outbreaks [41, 42]. That outbreak caused significant financial damages, estimated in billions of dollars [43]. Concerns regarding potential public panic and financial and other damages to the city that may be caused by sampling transportation systems may, thereby, detract city officials from approving microbiome surveys.

However, public officials should be made aware that sampling the transport system can help monitor and fight the spread of pathogenic microorganisms, particularly during seasonal outbreaks, and develop strategies that the public can adopt to improve travelers’ health, alleviate their concerns [44], and promote the use of public transport. Thus far, the majority of the bacteria identified in transit systems were benign commensal species typically found in our environment and skin [16]. In a survey of New York subway systems, Afshinnekoo et al. [2] found 1688 non-pathogenic bacteria species mostly associated with the skin flora. Only 31% of the species were identified as potentially opportunistic bacteria that, although possibly linked to diseases in people with compromised immune defense, are unlikely to be pathogenic in healthy individuals. The viruses found in the NY subway system were also generally harmless, as most belonged to the bacteriophages group that only infect bacteria [45]. We note, however, that 48% of the DNA belonged to unidentified organisms, which likely follow the proportion of other kingdoms of life that were identified: bacteria (46.9%), eukaryotes (0.8%), viruses (0.03%), archaea (0.003%), and plasmids (0.001%) [2]. Finding antibiotic resistance species, not unexpectedly, is also unlikely. Only 8% of the hand-touch surfaces in busses, trains, stations, hotels, and public areas of a hospital in central London contained methicillin-sensitive *Staphylococcus aureus* and no sites grew methicillin-resistant *S. aureus* (MRSA) [46]. A slightly higher percentage (28%) of bacteria cultured from the surfaces of NYC subways showed resistance to standard antibiotics [2]. In both studies, antibiotic resistance was defined by phenotypic assays. However, even when pathogenic organisms are found, the majority of infections can be avoided by washing hands with soap and water [47]. Notably, Afshinnekoo et al.’s [2] survey of the NY subway system was followed by a highest-ever peak in the number of users of the transit system [48, 49].

### Creating health risks to investigators

Generally, the risk for infection during sampling is considered low, and no greater than typical everyday life. However, sampling specific sites, such as sewage systems, public toilets, or animals, may carry risk for investigators. For instance, sampling animals may put the investigator at risk for zoonotic disease transmission, and wastewater is known to contain viable human pathogens [50–52]. In addition to sampling animals and wastewater, taking samples from hospitals may result in exposure to antibiotic-resistant bacteria, such as *MRSA* or *Pseudomonas aeruginosa* [53], yet the risk of actual infection exists mostly for immunocompromised people and not necessarily the scientists taking samples [54]. Nonetheless, to minimize the risk of exposure, investigators should adopt standard safety procedures such as not being in close proximity to potentially contaminated surfaces and animals. When sampling hospitals or public transport systems, close contact with people should be avoided. Hand washing can also significantly reduce the risk of potential infections [47]. Wearing safety equipment, such as face masks and gloves, protects workers from potentially harmful microorganisms [55]. The proper equipment should be selected based on the estimated risk level of the studied site. For example, the National Institute for Health and safety has a document recommending different types of masks, based on the potential hazard and professional judgment [56].

### Disparaging cities and public sites

Similar to as discussed above, the public’s association between microbiology and disease may result in a negative association for microbiome sampling sites. It is not uncommon to find reports of toxins and small particles [57] alongside possible pathogenic microorganisms [58] living in solid surfaces or the air, which arguably, causes public anxiety. However, historical precedence can be misleading in this case. In modern cities, gaining information about microbial populations will enhance cities’ efforts to improve public health [59]. We thereby suggest that microbiome studies will ultimately improve cities’ reputation via the public’s perception of greater public health monitoring.

Research efforts targeted at the indoor environment where most people spend ~ 90% of their time have significant potential to improve public health. The linkages between dust, microorganisms, and diseases such as asthma and allergies are established but generally poorly understood [60, 61]. It has been shown that dust collected from air-conditioning filters had high level of potentially harmful gram-negative bacteria [62]. Air-conditioners and ventilation systems may also contain bacteria, such as *Mycobacterium tuberculosis*, that can contribute to the bad quality of the air in apartments and buildings [62–64]. Hence, knowledge acquired about the pathogens in our immediate surrounding can also support efforts to improve the hygiene in public sites and reduce the risk of disease spread [65]. Ongoing public sampling and monitoring has an important role in alleviating existing fears of toxicity and pathogenicity.

### Stigmatizing healthcare facilities as healthcare hazards

The problem of disease dissemination in healthcare facilities has existed since their establishment. Hospitals are the ideal environment for the spread of pathogenic bacteria, as both patients and health workers are in contact with contaminated surfaces, immune-compromised patients, and each other. One in 25 US hospital patients develops a nosocomial infection [66]; of those, one in 10 dies from their infection. Similarly, in Europe, 3.8% of general ward and 15.3% intensive care unit patients acquired at least one nosocomial infection during their visit [67]. In some cases, patients are being advised to limit their hospital visits not only because the departments are busy [68] but because patients are at risk of contracting harmful infections [69–71]. These issues have already shaped the image of healthcare facilities as incubators that facilitate the creation and spread of antibiotic-resistant “superbugs” [72], and they are more acute in unhygienic institutions and those forced to release sick patients due to overcrowded departments, chiefly during seasonal outbreaks [73, 74]. Even sinks for hand washing have been recognized in aiding the spread of antibiotic-resistant bacterial pathogens [75]. It is now widely accepted that surfaces such as door handles, seats, and even floors are contaminated and may facilitate the rapid acquisition of antimicrobial resistance (AMR) [76] and transmission of several pathogens such as MRSA, Vancomycin-Resistant *Enterococci* (VRE), and norovirus [77–80].

While microbiome hospital surveys may reinforce this image, they are a crucial step to find a solution to this problem. For example, public microbiome projects like the resistomap [81] have been valuable to understand the spread of AMR. Hospital sampling also allows understanding how antibiotic resistance spread over time and space, which allows healthcare practitioners to focus efforts on preventing contamination [82]. Therefore, the potential benefits in improving that treatment and the well-being of patients [83] outweigh the potential concerns regarding hospital swabbing. Such monitoring projects are currently underway [17, 84, 85].

In addition to informing control of AMR and primary pathogens, microbiome surveys and monitoring efforts may inform the control of opportunistic pathogens. For example, *Legionella* grow in building plumbing systems and have emerged as a significant liability and public health concern for hospitals. Multiple strategies, such as on-site disinfection, are employed to control these opportunistic pathogens [86]. As these pathogens grow within a complex microbial ecology, microbiome monitoring has been proposed to inform their control [87].

### Encouraging fears of water safety

The increasing international demand for bottled water underscores a declining trust in water safety; indeed, health concerns are listed as one of the chief drivers of this demand [88]. Microbiological contamination has long been a threat to water safety. This was first recognized by Dr. John Snow after the cholera outbreak in 1854 in central London, which took the lives of more than 500 people, and after which there was a new found awareness that cholera and other pathogens could be spread through the drinking water [89]. For these reasons, water facilities in the UK and many other countries are constantly monitored and tested for pathogens, toxins, or other forms of contamination to limit potential public health impact [90].

Such monitoring, however, generally excludes sewage conveyance and treatment systems that are known to contain human and animal pathogens [50–52]. Moreover, not all public water sources around the globe are being monitored regularly, or at all. Therefore, monitoring pathogen presence and viability in water facilities is essential to evaluate infectious risk and prioritize the water sources that require increased monitoring [90]. This is essential to provide more comprehensive protection of the public’s safety and dynamic response to variegated risk across a city’s sources of water. Efforts informed by microbiome surveys are already underway in various sites to improve pathogen detection methods for water monitoring [91, 92].

### Perpetuating privacy and confidentiality fears

As privacy is becoming a growing concern, there is an increasing awareness of the risks of sharing information online. Paradoxically, though hacking to obtain biological information is extremely rare compared to other forms of hacking, the public is very conscious of privacy invasion associated with biological data [93]. While individual human identification from microbiome samples has yet to be achieved, some of the privacy concerns are valid. Fingertip microbial communities can show what keys of a computer keyboard were used and how recently, with traces being identifiable for up to 2 weeks at room temperature [94]. Lax and colleagues [85] showed that the microbiome of patients became more similar to their room microbiome the longer they stayed there. In other words, the room microbiome can be forensically analyzed to trace its inhabitants, but not only them. People have their own individual microbial “cloud” made up of biological particles emitted at a rate of ~ 10^6^ per hour [95]. These airborne bacterial emissions contribute to the settled particles around people and can potentially be used to identify individuals or those who came in contact with the person [96]. It is therefore conceivable that sampling of crime scenes and suspects for their microbiome will become a forensic utility in the future.

While privacy risks are realistic, the field of microbial forensics today is still in its infancy, and the dynamic microbial nature poses great challenges that may question the usefulness of microbial-based tool for forensics. Franzosa et al. showed that individuals who supplied their microbiome from skin and stool samples as well as their genetic code could be identified with an 80% accuracy based on their stool sample; however, the accuracy dropped to 30% when microbiome from other sites of the body was sampled, such as skin and mouth [97]. This study and others indicate that it is potentially possible to match an individual with their microbiome; however, the microbiome data are likely to be used in conjunction with other data such as DNA profiles because microbiome composition could be influenced by several factors such as cosmetics, antibiotic use, and general state of health [98].

Another chief concern is that researchers will be able to infer information about the individual’s health, habits, and lifestyle from their microbiome profile, which may then be accessed by third parties. This concern may also become realistic in the future when the microbiome can be harnessed to accurately trace the recent historical whereabouts of people [99]. Remarkably, microbiome privacy advocates have already released devices that can remove (enzymes) or replace (oligos) the DNA cloud that we leave behind [100].

To address all these concerns, privacy and safety measures should be applied to the collection and storage of microbiome data [101], and laws like the Genetic Information Nondiscrimination Act (GINA) of 2008 should be updated to include microbiome data. Such laws were created to protect personal information gathered from research involving collection of human data; however, in the case of GINA, non-human information is not protected. Since microbial DNA data are commonly stored in publicly available databases, there is a risk of identification even when the data are “anonymized” [102]. Misconceptions concerning anonymization and using meta-data resemble those made in the early genomic era, as human genomic data and their annotation were readily available online [103]. Previously, such a shift in data accessibility (dbGAP) has been put in place by the NIH and only after it became possible to extract information about individuals based on their genetic data [104]. Proper security measures should thereby be applied to microbiome data to prevent them from becoming a privacy risk.

### Raising new ethical questions

The huge leap in microbiome research enabled by rapid sequencing technologies has resulted in the development of large databases where microbial samples from humans and the environment are stored. These collections raise many questions [105] concerning the ethical and social implications of sampling the human microbiome. The two most contested subjects are returning the results to participants and informed consent.

To address the first subject, we first have to formulate the ethics that govern the microbiome by deciding whether microorganisms are parts of our body or separate entities. Although they are clearly inter-connected and exist as dynamic, continually exchanging ecosystems, legally, they are often treated differently. If the microbiome is separate from tissues, humans may have fewer rights to their own microbiome than to their tissues that harbor it. There are several reasons why the decision is difficult. First, due to the infancy of human microbiome studies, much of the data remain uncharted, hard to interpret, and/or unmappable to known genomes. Second, encountering or searching for pathogenic agents raises questions as to whether the findings should be reported to patients or public health authorities [106], since the relationship between bacterial colonization and infection is not yet clear. Moreover, there is little clinical validation of microbiome results linking to health or disease [107]. It can therefore be argued that it is unethical to report any scientific findings back to participants, absent a clear indication and validation.

The second debatable subject deals with what informed consent should encompass, particularly in the absence of regulation on microbiome data. Currently, many countries including the UK and USA have laws that protect human subjects by requiring a full disclosure of any potential risk and benefits in participation [108]. In the UK, such laws fall under the remit of the NHS ethics review procedure (i.e., mainly research involving patients). However, as our understanding of the human microbiome grows, the laws involving data protection should be revised and the requirements from researchers should be clarified. For instance, the use of extensive 15-pages consent forms written in extensive verbiage, such as those used by the human microbiome project (HMP), has been criticized [109]. It has been proposed that a shorter version of the official consent documents should be produced to maximize the amount of crucial information the subject is expected to comprehend [110, 111].

### Demonizing microorganisms

The idea that all bacteria are harmful and should be exterminated is substantially incorrect “common knowledge,” yet various products are promoted in the popular media that “kill 99.9% of all bacteria” [112]. Although we are surrounded by information sources urging us to get rid of bacteria as a source of morbidity and mortality, most bacteria are harmless and, often, beneficial [113]. Of the many bacteria that colonize our skin, nasal passages, and colon generally positively contribute to our well-being. The bacteria in our environment also influence our health and well-being [114–117]. Diverse colonies of bacteria live in symbiosis with our body and are essential for the healthy functioning of multiple bodily systems, like the GI tract. Disruption in the gut microflora may cause irritable bowel disease (IBD), characterized by a continuous inflammatory process in the gut, even after the primary pathogen has been eradicated [118]. Further investigations into how to stabilize and perhaps diversify our microenvironment may improve our quality of life [119].

Bacteria are also notoriously associated with dirt, diseases, and a general state of uncleanliness and struggling with the poor public relations of bacteria can be expected to hinder public microbiome initiatives at various levels. Nonetheless, similar to the complex interactions of commensal, opportunistic, and pathogenic bacteria of our internal microbiome, the bacteria of our external environment also have symbiotic relationships with each other and us [4]. Indoor microbial communities have been shown to significantly differ from the multiplicity of the outdoor microbiome described thus far [120]. The indoor communities are affected by the selective pressures of the environment, such as location, ventilation, and the presence of other humans. Students in a classroom increase the bacterial load of the air (by two orders of magnitude) when compared to an empty classroom [121]. Several studies have implied that a certain diversity and amount of bacteria in our indoor environment is beneficial and may prevent the development of illnesses like asthma in early stages of life [114–116]. These studies, though limited in size, offer a new angle to consider chronic illnesses and encourage a public’s reconsideration of the value of bacteria and invite further research in this field.

## Recommendations

Based on our long-term experience with public engagement [22, 122], we make several recommendations that can enhance the accessibility and transparency of microbiome research. Establishing a website that outlines the hypotheses, goals, and findings of the study would make a useful resource of information. Business cards with the website address, the purpose of the work, and its implications handed out to curious bystanders would mitigate anxiety and allow quick dissemination of the research data. Investigators should consider carefully how their activities and results can be misinterpreted [123] and avoid grades and labels. Keeping a live blog of the website designed for the broader audience would allow the team to announce upcoming steps and also share the experience of interacting with the public [124]. Such a platform will allow the researchers to promote the study on social media (Facebook and YouTube) as well as on various forums and encourage dialog between researchers, participants, and the general public [125].

We note that several guidelines for public engagement are already in place. For instance, the Responsible Research and Innovation (RRI) is an initiative by the European Union working toward an open science and innovation system that ultimately tackles societal changes [126]. It promotes the active engagement of key stakeholder groups (for example, members of the public, representatives of relevant interest groups, and leaders of relevant organizations), from the earliest stages of a project in order to ensure that the research is designed in close consultation with them and takes into account their questions and concerns. This could be a useful approach for researchers wishing to undertake microbiome studies to adopt existing guidelines set by these organizations.

Therefore, meeting with decision makers in the relevant organizations to secure their support of the project is a recommended step to keep the public representatives informed of ongoing research, as is now done with the City Council in NYC. This may also yield fruitful collaborations, as science can be linked with politics to increase the public outreach [127]. An example of public engagement could be delivering short tutorials for school children on the importance of washing hands and improving the overall hygiene in schools. Also, such engagement enables the teaching of emerging aspects of microbiome and metagenomics research, including epigenetics [128], extremophiles [129], and even studies of microbiomes in space [130]. Finally, it is important to communicate and evaluate the risks of identification from microbial samples and the accidental human DNA collected in the process to the public.

## Conclusions

Genome-enabled technologies created a dramatic increase in our ability to study the microbiome in various environments and hosts including our, largely uncharted, indoor and outdoor environments. The insights gained from this research may substantially alter our prior perceptions on microorganisms and their effect on our lives and health. While the public has shown an interest in projects aiming to chart the gut microbiome of humans [131] or animals [132] and even test the microbiome behavior in space [7], there remain concerns that microbiome mapping of the open space environment would raise major public concerns, reservations from using public facilities, and social unrest. To make such research possible, it is imperative that scientists would understand these risks, develop research projects that mitigate them, and report the results in a responsible, transparent, and accurate manner.

## Abbreviations

AMR: Antimicrobial resistance
GINA: Genetic Information Nondiscrimination Act
MetaSUB: Metagenomics and Metadesign of Subways and Urban Biomes
MRSA: Methicillin-resistant *Staphylococcus aureus*
VRE: Vancomycin-Resistant Enterococci
